# Retraction: Influenza or not influenza: Analysis of a case of high fever that happened 2000 years ago in Biblical time

**DOI:** 10.1186/1743-422X-7-190

**Published:** 2010-08-13

**Authors:** Kam LE Hon, Pak C Ng, Ting F Leung

**Affiliations:** 1 Department of Paediatrics, The Chinese University of Hong Kong, Prince of Wales Hospital, Shatin, Hong Kong SAR, China

## 

The Editor-in-Chief of *Virology Journal *and the authors of this article [1] acknowledge that the article does not provide the type of robust supporting data required for a case report and that the observations made are mostly speculative. As such, the article does not fulfill the criteria for a case report and does not meet the high standards expected of the journal. The authors and the Editor-in-Chief have therefore agreed to retract the article.
